# Efficacy of treating bacterial bioaerosols with weakly acidic hypochlorous water: A simulation chamber study

**DOI:** 10.1016/j.heliyon.2024.e26574

**Published:** 2024-02-22

**Authors:** Saowanee Norkaew, Sumiyo Narikawa, Ukyo Nagashima, Ryoko Uemura, Jun Noda

**Affiliations:** a Faculty of Public Health, Thammasat University, Khlong Nueng, Klong Luang, Pathum Thani, 12121, Thailand; b Research Unit in Occupational Ergonomics, Thammasat University, Khlong Nueng, Klong Luang, Pathum Thani, 12121, Thailand; c School of Veterinary Medicine, Rakuno Gakuen University, Bunkyodai-Midorimachi, Ebetsu, Hokkaido, 069-8501, Japan; d Department of Veterinary Sciences, Faculty of Agriculture, University of Miyazaki, GakuenKibanadai-Nishi, Miyazaki, 889-2192, Japan

**Keywords:** Weakly acidic hypochlorous water, Bactericidal effect, Gram-positive/negative bacteria, Bioaerosols, Biofilm, Fomite, Survival rates

## Abstract

The COVID-19 pandemic highlighted the dangers of airborne transmission and the risks of pathogen-containing small airborne droplet inhalation as an infection route. As a pathogen control, Weakly Acidic Hypochlorous Water (WAHW) is used for surface disinfection. However, there are limited assessments of air disinfection by WAHW against airborne pathogens like bioaerosols. This was an empirical study evaluating the disinfection efficacy of WAHW in an atmospheric simulation chamber system against four selected model bacteria. The strains tested included *Staphylococcus aureus* (SA), *Escherichia coli* (EC), *Pseudomonas aeruginosa* (PA), and *Pseudomonas aeruginosa* (PAO1). Each bacterial solution was nebulized into the chamber system as the initial step, and bioaerosol was collected into the liquid medium by a bio-sampler for colony forming units (CFU) determination. Secondly, the nebulized bacterial bioaerosol was exposed to nebulized double distilled water (DDW) as the control and nebulized 150 ppm of WAHW as the experimental groups. After the 3 and 30-min reaction periods, the aerosol mixture inside the chamber was sampled in liquid media and then cultured on agar plates with different dilution factors to determine the CFU. Survival rates were calculated by a pre-exposed CFU value as a reference point. The use of WAHW decreased bacterial survival rates to 1.65–30.15% compared to the DDW control. PAO1 showed the highest survival rates and stability at 3 min was higher than 30 min in all experiments. Statistical analysis indicated that bacteria survival rates were significantly reduced compared to the controls. This work verifies the bactericidal effects against Gram-positive/negative bioaerosols of WAHW treatment. As WAHW contains chlorine in the acid solution, residual chlorine air concentration is a concern and the disinfection effect at different concentrations also requires investigation. Future studies should identify optimal times to minimize the treated time range and require measurements in a real environment.

## 1 Introduction

The COVID-19 pandemic highlighted the risk of airborne transmission and dangers of small airborne droplet inhalation as an infection route [1]. Poor indoor air quality (IAQ) became an important concern during the spread of the coronavirus disease. The Environmental Protection Agency (EPA) reported that people in industrialized countries spend over 90 % of their time in an indoor environment [2]. In modern society, we spend a large amount of time in indoor environments resulting in airborne infection becoming a significant danger and making indoor air quality an increasingly important health issue. Throughout a person's lifetime, they are exposed to aerosols, omnipresent in indoor air. Consequently, the effect of indoor aerosol exposure ranges from insigniﬁcant to fatal, depending on aerosol composition and concentration, type of indoor environment, duration of exposure, age, gender, susceptibility, and other factors [[3], [4], [5], [6]]. Notably, improper building ventilation leads to “sick building syndrome” and other illnesses [7,8]. Additionally, maintaining indoor air quality is increasingly difficult due to economic growth and urban development, with increased urban population density, and the construction of high-rise buildings, schools, and offices with more air-tight structures. Due to the prevalence of energy-efficient buildings with higher atmospheric integrity, indoor air quality faces challenges in the effective use of active ventilation systems. Indoor air quality may decrease partly due to energy conservation strategies promoting air-tight structures with active ventilation. However, these active ventilation systems rely on house owners using the active ventilation. As energy costs increase, some residents reduce active ventilation rates, reducing air exchange rates and lowering indoor air quality. Other potential factors for air quality reduction include indoor emissions from the many new materials, especially polymers and numerous electronic devices [9]. Moreover, construction materials, home decoration materials, pesticides, and cleaning chemicals all contribute to poor indoor air quality [[9], [10], [11]]. Higher air pollution levels are observed in densely inhabited areas. Road transport is one contributor to this, with vehicle emissions pollutants including carbon monoxide, nitrogen dioxide, sulfur oxides, and particulate matter [[12], [13], [14]]. Previous studies found vehicle-related emissions, such as NO_2_, O_3_ and PM_2.5_ occurring in high concentrations in high-rise building/city areas [15,16]. Exposure to these pollutants while indoors may be a large fraction of the integrated daily exposure to gas, fume, and particles (especially UFP: ultraﬁne particles and PM: Particulate matter), strongly depending on area activities, source events, and site speciﬁcity. Previous studies focused on chemical substances with gas and particulate phases, whereas urban area air pollution may increase the presence of airborne microorganisms, including pathogens, thus increasing infectious disease cases [[17], [18], [19]]. Previous studies reported that the presence of chemical and biological pollutants affected people's health, with all reports agreeing that chemical and biological pollutant accumulation indoors leads to significant adverse health effects. Indoor pollutants are constituted by emissions from building and materials, cleaning and personal products, cooking, environmental tobacco smoke, human metabolism, and other biological sources such as mold, pets, and house dust mites [20]. Bio-aerosols, as a biological pollutant, are usually defined as airborne bacteria, fungi, viruses, pollen, and their byproducts. These biological pollutants are associated with a wide range of health effects in indoor air environments [21]. Improved exposure and risk assessment methods are needed, together with a focus on exposure control, to reduce the severity and risks associated with human exposure to indoor-outdoor pollutants, including bioaerosols [22].

Reducing levels of airborne pathogens such as bacteria, viruses, and fungi is an important goal for air quality management [23]. Sufficient ventilation is recommended practice to maintain a safe environment, and is improved with disinfectant usage to improve safety in the indoor environment. There are different methods for minimizing the growth of biological entities. Among various methods, silver metal particles, transition metal complexes, and photo-catalysts may enhance the degradation of pathogenic microorganisms to minimize the risk of infection [[24], [25], [26]]. Weakly Acidic Hypochlorous Water (WAHW) is an appealing environmentally-safe disinfectant due to its low residual properties, independence to water hardness, and common use as a surface disinfectant [[27], [28], [29]]. Hypochlorous acid is an endogenous substance in all mammals. The mechanism of hypochlorous acid disinfection involves the destroying of the cell wall of microbes which allowed the disinfectant to inactivate the microorganism [30,31]. However, the assessment of air disinfection by WAHW against bioaerosols is limited. The objective of this study was to apply WAHW treatment in an atmospheric simulation chamber system against four selected viable model bacteria to clarify its disinfection efficacy. An additional study was carried out on PAO1, possessing a higher biofilm formation capacity, further examining the hypothesis that biofilms act as fomites, increasing WAHW resistance. This may explain how pathogens, in general, may evade atmospheric stresses and prolong airborne viability.

## 2 Materials and methods

### 2.1 Study site and study design

The study was conducted at the School of Veterinary Medicine, Rakuno Gakuen University, Hokkaido, Japan, from November 2022 to January 2023. An experimental study investigated the effectiveness of treating bacterial bioaerosols with weakly acidic hypochlorous water (WAHW) in a simulation chamber. Air samples were collected to estimate the bacterial survival rates. The differences between the control and experimental groups after 3 min and 30 min from the initial measurements were made, and a *p*-value of <0.05 was considered statistically significant.

### 2.2 Chamber experiment

**Fig. 1:**
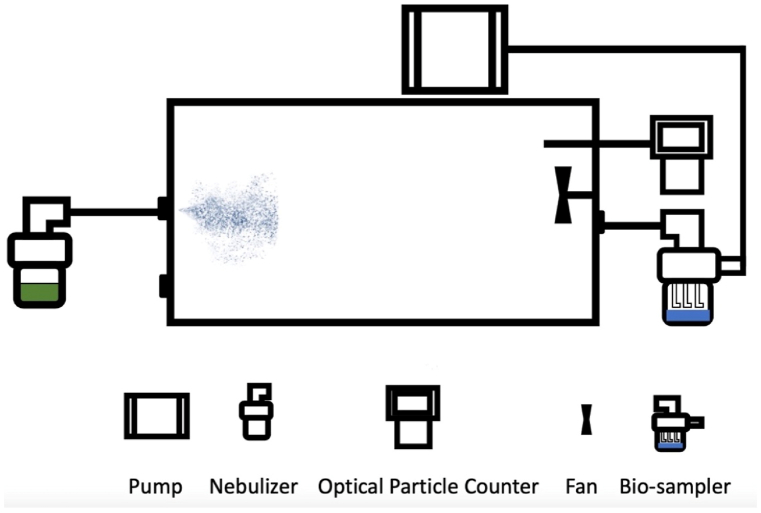
The chamber system.

A reaction chamber system was employed to examine the survival of bacterial bioaerosols. The chamber was used to investigate the viability rates (survival) of bioaerosols of three bacterial species. The external dimension of the chamber is 40(D) × 40(D) × 100(H) cm and, the total volume is 128 L [32]. Humidity and temperature in the chamber were examined in all experiments to exemplify the ability of an experimental system to provide a stable within-chamber environmental condition. Temperatures ranged from 22.3 to 24.3 °C and relative humidity ranged from 41.1% to 59.7%. Average and standard deviation (SD) of temperature and relative humidity during this period were 23.3 ± 0.7 °C and 47.9 ± 5.2%, respectively. Fig. 1 indicates a 128 L Teflon chamber system.

### 2.3 Model bioaerosols

The strains used in the chamber experiments were *Staphylococcus aureus* (ATCC29213), *Escherichia coli* (DH5α), *Pseudomonas aeruginosa* (ATCC27853), and *Pseudomonas aeruginosa* (PAO1: ATCC15692). Two days prior to the start of the experiment, each strain was inoculated into Mueller-Hinton agar and incubated at 37 °C for 24 h. All bacteria solutions were prepared in phosphate buffer solution (PBS), and were higher than 10^8^ colony forming unit (CFU) per ml.

### 2.4 Aerosol generation

A compressor nebulizer was employed to generate bioaerosols, which method was adopted from Ueki et al. and Noda et al. [[32], [33], [34]]. A compressor-type nebulizer (NE-C30, OMRON CO., Ltd., Kyoto, Japan) was used both to generate bioaerosols and the introduction of WAHW. The sprayed rate was ca. 0.35 ml/min. Weak Acid Hypochlorite Water (WAHW) containing 150 ppm free chlorine was prepared by diluting a 200 ppm WAHW solution with double distilled water (DDW) produced with equipment (Yamato Scientific Co., Ltd, Tokyo, Japan) on the day of use. The DDW produced with the same equipment was used for the control group, which was also prepared on the same day of the experiment.

### 2.5 Aerosol sampling from the chambers

According to the microorganism inactivate time, WAHW decontaminating effectiveness and bioaerosols ability to stay as an airborne for an extended period before they settle on environmental surfaces or enter the respiratory tract, the sampling time techniques were adopted from previous studies (Block & Rowan, 2020; Unterwurzacher et al., 2021). The prepared bacterial solution was placed in a nebulizer and ca. 1 ml of the solution was sprayed into the chamber for 3 min. The nebulized bacterial aerosols in the chamber were allowed to diffuse and stabilize for 3 min, and particle size and number measured by Optical Particle Counter (OPC: Aerotrack 9306-V2, TSI, Shoreview, MN, U.S.A.). The aerosol in the chamber was collected by a BioSampler® (SKC, Eighty Four, PA, USA) containing 14 ml of PBS over 1 min with a flow rate of ca. 10 L/min. The BioSampler functions similarly to an impinger, condensing aerosols into a liquid phase by pumping air through three angled nozzles, vortexing aerosols to increase sample collection efficiency. Similarly, DDW was sprayed as the control and the same procedure followed. Collections of DDW (control) and WAHW (experiment) were made after 3 min and 30 min of spraying. After sampling, the liquid solution was transferred to a sterile test tube. The collected liquid was transferred to a centrifuge tube (15 ml) and centrifuged at 3600 rpm/1884G/min over 3 min to concentrate the bacteria at the centrifuge tube bottom section, then the supernatant was discarded. A 100 μl aliquot of the centrifuge tube concentrate was inoculated onto the Mueller-Hinton agar medium. The medium was incubated at 37 °C for 24 h, CFU measurement was performed, and the following equations [1,2] were used to calculate survival rates.

Control group:

(1) Survivalrate[%]=(CFUAfternebulizingDDW/CFUBeforenebulizingDDW)×100

Exposure group:

(2) Survivalrate[%]=(CFUAfternebulizingWAHW/CFUBeforenebulizingWAHW)×100

### 2.6 Particle counting

Particle measurements were conducted using the OPC (Aero Trak Model 9306-V2, TSI, USA). Prior to each experiment, the chamber system was air-purged through a High-Efficiency Particulate Air (HEPA) filter unit (Pall, NY, U.S.A.). After the purge, the chamber system was monitored by OPC to confirm particle-free conditions.

### 2.7 Biofilm observation

*Pseudomonas aeruginosa* (PAO1 and ATCC27853) were used to assess biofilm protection while in the aerosol form. In this study, the classical quantification technique of violet staining was used to measure the biofilm formation ability, a method adapted from Ref. [35]. To examine bacterial strain-dependent biofilm formation capability in the LB broth liquid media, three glass slides were immersed in each bacteria solution and incubated at 37 °C for 15–48 h. Prior to the experiment, the liquid media with bacteria were cultured for 6 h to almost equal bacteria concentrations. After that, three slides were immersed in two LB broth-bacteria solutions with further incubation. One slide each from each batch was removed from the solutions after 15, 24, and 48 h. Finally, the cultured bacterial cells on the slides were stained with crystal violet assay (0.1%) solution added to each slide and carefully washed under running tap water to remove excess crystal violet dye. Then, slides were tapped on paper napkins to remove excess liquid and air-dried for the photographic record.

### 2.8 Statistical analysis

Two-way ANOVA was used to determine statistically significant differences between control and experimental group and effected of time at 3 min and 30 min stabilization with a *p-*value of <0.05 considered statistically significant.

## 3 Results

### 3.1 Survival rate

**Fig. 2:**
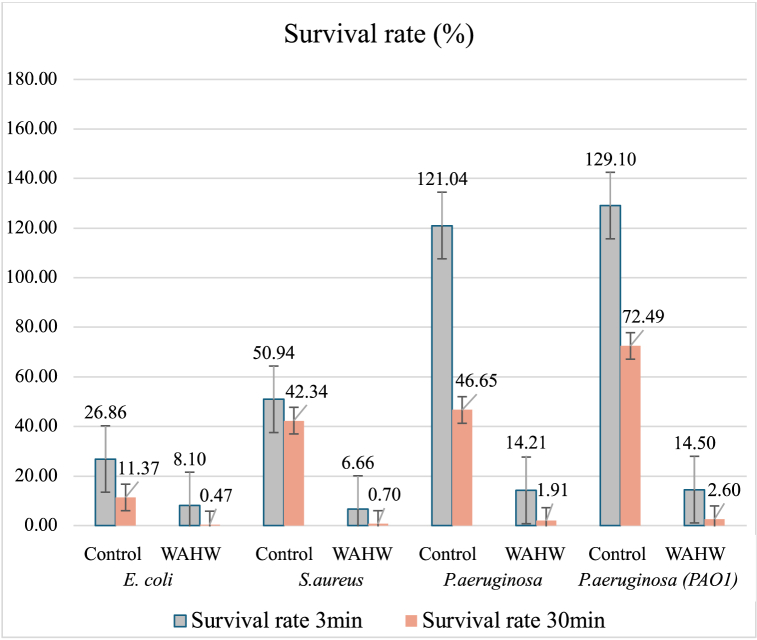
Mean and survival rates trends for four types of bacteria.

In this study, the 4 strains’ survival rates and reduction rates in the chamber experiments are listed in Table 1. The survival rate ranged between 0.47 and 129.10%, with the highest observed for *P. aeruginosa* (PAO1: ATCC15692). The survival rate order in the chamber by CFU measurement was *Pseudomonas aeruginosa* (PAO1: ATCC15692), *Pseudomonas aeruginosa* (ATCC27853), *Staphylococcus aureus* (ATCC29213), and *Escherichia coli* (DH5α). Comparing survival rates between the control group (Double distilled water spray) and the experimental groups (WAHW spray) showed that each bacteria strain had better survival in the control group than the experiment group. Moreover, the survival rate trend over 3 min of stabilization was found higher than in bacteria after 30 min. However, the survival rate at 3 min for *Pseudomonas aeruginosa* (ATCC27853) and *Pseudomonas aeruginosa* (PAO1: ATCC15692) ranged between 121.04 and 129.10 % in the control group and 14.21–14.50% in the experimental group. Furthermore, after 30 min of stabilization in the control group, the highest survival rate was found for *Pseudomonas aeruginosa* (PAO1: ATCC15692) with 72.49 %, while the 3 other bacteria strains were lower than 50% survival. The exposure to WAHW yielded survival rates for each type of bacteria lower than 15%, ranging from 0.47% to 14.50%. The rate reduction indicates the normalized experimental groups' survival rates based on the control groups' survival rates within the same bacterial species. The mean and trend of survival rates in four types of bacteria are shown in Fig. 2.

**Table 1:** The survival and reduction rates of each bacteria in control and experiment groups are indicated.

Type of Bacteria	Group	Survival/Reduction rates (%)		*p*-value*	*p*-value**
		3min	30min		
*E. coli*	Control	26.86	11.37	<0.001*	<0.001*
	Experiment	8.10	0.47		
	Reduction	30.15	4.13		
*S.aureus*	Control	50.94	42.34	<0.001*	0.067
	Experiment	6.66	0.70		
	Reduction	13.07	1.65		
*P.aeruginosa*	Control	121.04	46.65	<0.001*	0.01*
	Experiment	14.21	1.91		
	Reduction	11.74	4.09		
*P.aeruginosa* (PAO1)	Control	129.10	72.49	<0.001*	<0.001*
	Experiment	14.50	2.60		
	Reduction	11.23	3.59		

**Note**: *p*-value*: effect of treatment groups (Control vs. WAWH), *p*-value**: effect of time points (3 min vs. 30 min), NA: Not Applicable.

Two-way ANOVA was used to compare the mean CFU difference between the control and experiment group. Statistically significant differences were observed for all selected bacteria between the control and experimental (*p*-value <0.05). Similar to the effect of stabilization time, *E. coli*, *P. aeruginosa*, and *P. aeruginosa* (PAO1) yielded statistically significant differences between time (*p*-value <0.05), while *S. aureus* showed no statistically significant difference in mean between time (*p*-value = 0.067). These results indicated significant differences (*p*-value <0.05) due to the treatment. The results are reported in Table 1.

### 3.2 Particle concentration

**Fig. 3:**
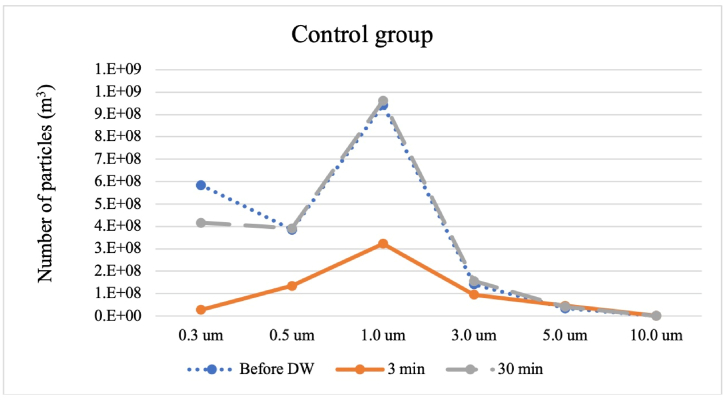
Particle count before DDW, 3 min, and 30 min after control group (DDW) spray.

**Fig. 4:**
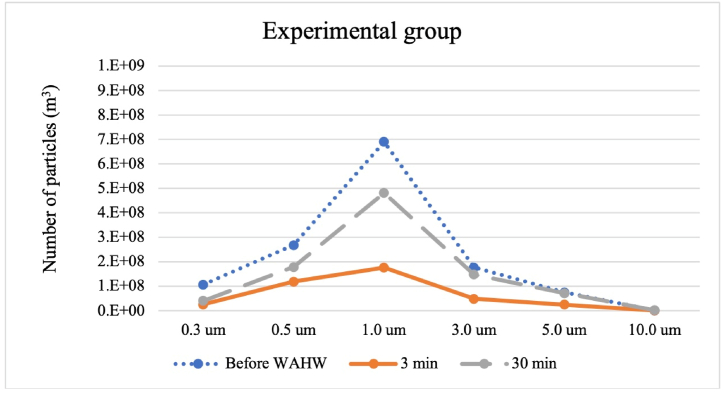
Particle count before WAHW, 3 min, and 30 min after experiment group (WAHW) spray.

Fig. 3, Fig. 4 indicates the aerosolized PAO1 particle numbers and size distribution information. The order of the PAO1 in the chamber due to particle count of the present study was before DDW/WAHW, after 30 min and 3 min spayed shown for both control and experiment groups. The results demonstrate that the WAHW group yielded lower particle numbers than the DDW group. However, the 1.0 μm particle size was observed at the highest concentration for each experiment including before DDW/WAHW treatment, after spraying at 30 min, and after spraying at 3 min in both DDW and WAHW groups. Meanwhile the concentration range of larger-sized particles (above 3.0, 5.0, and 10.0 μm) were similar and proportionally to time, reduced in all groups. The particulate concentration steadily decreases over time, and increases after a while (more true degradation with less re-suspension). The DDW control group showed greater fluctuation over time due to the direct effect of biofilm material absorbing water vapor and becoming stickier, interacting with the chamber wall. After 30 min, the drying effect released the particles attached to the chamber wall, reaching the same particle concentration level as before DDW addition. The DDW group did not affect the bacteria-biofilm mixture integrity, while the WAHW group degraded the bacteria-biofilm mixture, gradually decreasing particle number.

### 3.3 Biofilm observation

**Fig. 5:**
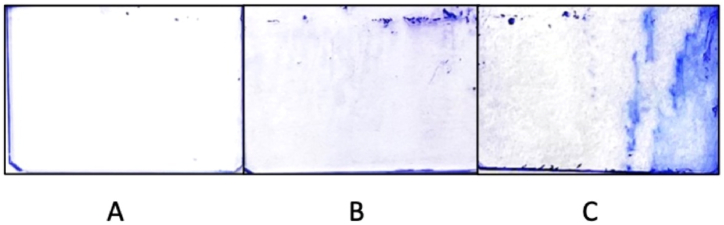
Biofilm observation of *Pseudomonas aeruginosa* (ATCC27853). Images of *Pseudomonas aeruginosa* (ATCC27853) biofilm growth over time at 15 (A), 24 (B), and 48 (C) h after seeding in a static culture.

**Fig. 6:**
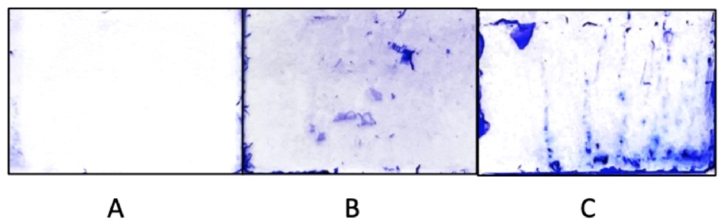
Biofilm observation of *Pseudomonas aeruginosa* (PA01). Images of *Pseudomonas aeruginosa* (PA01) biofilm growth over time at 15 (A), 24 (B), and 48 (C) h after seeding in a static culture.

*Pseudomonas aeruginosa* biofilms stained with crystal violet were observed by microscopy after different incubation times (15, 24, and 48 h), images are shown in Fig. 5, Fig. 6. Images of *Pseudomonas aeruginosa* ATCC27853 stained with crystal violet at different incubation times are shown in Fig. 5 and PAO1 in Fig. 6. For 15-h incubation time biofilms (Fig. 5, Fig. 6A), the biofilms were clear in both the ATCC27853 and PAO1 samples. For the 24-h incubation time biofilms (Fig. 5, Fig. 6B), biofilms formed leaving observable biofilm features. Furthermore, after 48-h incubation time, the biofilms (Fig. 5, Fig. 6C) were highly featured with an increased concentration. It may be that once a biofilm grows past the initial stage, further biofilms may grow upon older films. The employed method of visualization is somewhat limited as it cannot distinguish live cells from dead strains.

The *Pseudomonas aeruginosa* ATCC27853 and PAO1 solution from the LB broth after 48-h incubation and a step dilution in phosphate buffer solution (PBS) yielded between 46 and >1000 colony forming unit (CFU) per ml. The results shown that in *Pseudomonas aeruginosa* ATCC27853, at 10^3^ dilution was >1000 CFU, 10^4^ dilution was 395 CFU and at 10^5^ was 46 CFU. For PAO1 found more than 1000 CFU, 732 CFU and 74 CFU at 10^3^, 10^4^ and 10^5^ dilutions, respectively.

## 4 Discussion

The respective survival rate order in the chamber by CFU measurements was PAO1: ATCC15692, ATCC27853, ATCC29213, and DH5α. These results demonstrate that WAHW has promising bactericidal effects against Gram-positive/negative bioaerosols reflecting previous studies reporting that hypochlorous acid leads to induced thiol modification in Gram-positive bacteria [36,37]. The active chlorine component of WAHW is primarily HClO, which easily penetrates the lipid bilayers of microbial cell walls and cell membranes [38,39]. It is also faster-acting than other chlorine species and has a greater ability to kill pathogens by irreversibly denaturing key components, namely nucleic acids (DNA/RNA), mitochondria, and enzymes [40]. In this study, PAO1 demonstrated the highest survival rate, also presenting a higher capacity for biofilm formation among the examined bacteria, followed by *P. aeruginosa (*ATCC27853). Previous studies report a variety of abiotic stress response mechanisms of *P*. *aeruginosa*, including biofilm formation, and upon biofilm formation, bacteria become more resistant to antibiotic drugs, with no confirmed effective disinfection method [[41], [42], [43]]. Moreover, *P. aeruginosa* is a pathogen adapted to survive in chemically stressful environments, including exposure to reactive chlorine species [44]. Due to the ability of *P. aeruginosa* to form biofilms [43], and studies reporting that disinfectants are effective against *P. aeruginosa* [45], bacteria possessing biofilm formation capabilities, such as PAO1, demonstrate a resistance to disinfection, conferred by biofilms [43,46] contributing to bacteria survival rates.

In this study, Weak Acid Hypochlorite Water (WAHW) of 150 ppm was used, with the solution diluted from 200 ppm using distilled water. Despite 200 ppm concentration being an acceptable aqueous solution for disinfection in the EU (up to 200 ppm of oxidative titratable chlorine (Cl)), in the US (180–460 ppm) and the US by FDA (100–200 ppm) [47], previous studies reported low concentrations reducing viral titer in short reaction times [48], while HOCl at 100 ppm reduced the surface bacterial load in 20 min after application [30]. Nevertheless, the chlorine concentration effect against biofilm-forming *P. aeruginosa* is unreported [49,50]. Therefore, the effectiveness of WAHW observed in this study leads to a baseline for further evaluating appropriate concentrations to control biofilm forming bacteria.

In terms of time in longer the chamber, the survival percentage for stabilization for 3 min was higher than 30 min in all experiments. After longer bacterial stabilization periods, conditions become greatly different. Microorganisms are a major component of indoor dust particles [51], and airborne dust can settle on high contact fomites, such as door handles and other surfaces [52]. Thus, dry surface biofilms can transfer from fomite to fomite, becoming a persistent pathogen source and survive for more than a year due to biofilm-related desiccation protection [53]. Moreover, biofilm-forming bacteria are reported to have increased removal resistance against cleaning agents and disinfectants, as well as increased tolerance to chlorine [54,55]. Thus, resting time is important as bioaerosols may settle on chamber surfaces. Studies reported bacteria survival factors including temperature, pH, ultra-violet radiation, salinity, humidity, and nutrient availability [41,56,57]. By two-way ANOVA analysis, most tested bacteria were statistically influenced by experimental concentrations and stabilization time. Only *S. aureus* showed no significant difference in mean values between the 3- and 30-min sampling periods. Similarly, the particle counts show that for WAHW groups, lower particle numbers were observed than for DDW groups after 30 min. Fig. 3, Fig. 4 depict different particle concentration recovery trends before and after the nebulization of DDW and WAHW into the PAO1 bacterial bioaerosol-containing chamber. After 3 min, both mixtures decreased concentrations for the 0.5–3 μm size range. However, after 30 min reaction time, only the DDW group recovered to the previous particle size distribution in Fig. 3. The WAHW group did not recover to the same level as the DDW group, indicated in Fig. 4. This may explain why the DDW group has a relatively minor impact on the cell integrity of the PAO1 bacteria, with the particle concentration returning to pre-DDW nebulization levels. While, for the experimental group, WAHW damages bacteria physical integrity. Thus, after 30 min of nebulization, particle numbers are lower than before WAHW spraying. This may result from disinfectant effects, in which WAHW reduces the amount of PAO1 due to particle-size change. Several previous studies reported the effects of microbial characteristics, including treatment strategies [[58], [59], [60], [61]], and hydraulic conditions [62].

Moreover, not only biological hazards, but also chemical hazards must be considered as risk factors in a more integrated manner. Previous studies reported associations between environmental factors such as indoor air concentrations of carbon dioxide (CO_2_), carbon monoxide (CO), total volatile organic compounds (VOCs) (ppm), Nitrogen Oxide (NOx), Sulfur oxides (SOx), Particulate matters (PM), temperature (°C), relative humidity (RH%), and biological agents (bacteria and viruses) as contributing to “sick building syndrome”, reduced pulmonary function, respiratory problems including allergic rhinitis and/or asthma, along with cardiovascular disease, and/or cancer [[63], [64], [65], [66], [67], [68], [69]]. Moreover, according to World Health Organization (WHO) estimates, particle pollution contributes to approximately 7 million premature deaths each year, making it a leading cause of worldwide mortality [70]. Particularly contributing to respiratory and allergic diseases (including asthma, chronic obstructive pulmonary disease, pneumonia, and other infectious diseases such as tuberculosis), lung cancer risk and the nervous system [71,72]. A previous report evaluating ambient bioaerosols in China revealed an increase in airborne microorganism number in line with fine particulate pollutants emission [73]. To mitigate airborne health hazardous components, further study is required to understand the relationships between bioaerosols and particulate matter to impact the viability of airborne biological components. Prolonged bacterial bioaerosol viability due to newly generated soot is reported [74]. Also, field measurements indicated high bacterial diversity in long-distance transported dust, the dust acting as airborne fomites, carrying and preserving biological components in the air [75]. The interaction mechanism between airborne fomites and microbes requires further investigation. Thus, to maintain air quality and ensure health, a wholistic approach combining analysis of the chemical and biological components is required to effectively evaluate ambient air pollutants.

## 5 Conclusions

This study evaluated the disinfection efficacy of aerosolized WAHW using four model bacteria in an atmospheric simulation chamber. The aerosolized WAHW with available chlorine concentration of 150 ppm was confirmed to have bactericidal effects against Gram-positive and negative bacteria. The highest survival rate was for *Pseudomonas aeruginosa* (PAO1: ATCC15692) and the lowest survival rate was for *Escherichia coli* (DH5α). The order of highest to lowest survival rate in the chamber by CFU measurement was *Pseudomonas aeruginosa* (PAO1: ATCC15692), *Pseudomonas aeruginosa* (ATCC27853), *Staphylococcus aureus* (ATCC29213), and *Escherichia coli* (DH5α). Two-way ANOVA results revealed that all tested bacteria showed significant differences between control and experiment for stabilization time. The survival rates of *E. coli*, *P. aeruginosa*, and *P. aeruginosa* (PAO1) were significantly different between the two time levels, while *S. aureus* was not significantly different in these time levels.

This study focused on the effectiveness of the aerosolized WAHW against selected bacteria. As WAHW contains chlorine in the acid solution, residual chlorine air concentration is a concern. Future studies should include lowering WAHW concentration and other time series for bactericidal effect, which may identify optimal concentration and times to minimize the necessary WAHW amount and apply time range in a real environment.

## Data availability

All data underlying the findings described in this study are fully available without restriction.

## CRediT authorship contribution statement

**Saowanee Norkaew:** Writing – review & editing, Writing – original draft, Visualization, Validation, Investigation, Formal analysis, Data curation. **Sumiyo Narikawa:** Writing – original draft, Visualization, Validation, Investigation, Formal analysis, Data curation. **Ukyo Nagashima:** Writing – original draft, Visualization, Validation, Methodology, Investigation, Formal analysis, Data curation. **Ryoko Uemura:** Writing – original draft, Resources, Methodology, Funding acquisition. **Jun Noda:** Writing – review & editing, Writing – original draft, Visualization, Validation, Supervision, Resources, Project administration, Methodology, Investigation, Funding acquisition, Formal analysis, Data curation, Conceptualization.

## Declaration of competing interest

The authors declare that they have no known competing financial interests or personal relationships that could have appeared to influence the work reported in this paper.
